# Genome Wide Association Study Identifies New Loci Associated with Undesired Coat Color Phenotypes in Saanen Goats

**DOI:** 10.1371/journal.pone.0152426

**Published:** 2016-03-31

**Authors:** Pauline Marie Martin, Isabelle Palhière, Anne Ricard, Gwenola Tosser-Klopp, Rachel Rupp

**Affiliations:** 1 INRA, UMR 1388 Génétique, Physiologie et Systèmes d’Elevage, Castanet-Tolosan, France; 2 Université de Toulouse INPT ENSAT, UMR 1388 Génétique, Physiologie et Systèmes d’Elevage, Castanet-Tolosan, France; 3 Université de Toulouse INPT ENVT, UMR 1388 Génétique, Physiologie et Systèmes d’Elevage, Toulouse, France; CSIRO, AUSTRALIA

## Abstract

This paper reports a quantitative genetics and genomic analysis of undesirable coat color patterns in goats. Two undesirable coat colors have routinely been recorded for the past 15 years in French Saanen goats. One fifth of Saanen females have been phenotyped “pink” (8.0%) or “pink neck” (11.5%) and consequently have not been included in the breeding program as elite animals. Heritability of the binary “pink” and “pink neck” phenotype, estimated from 103,443 females was 0.26 for “pink” and 0.21 for “pink neck”. Genome wide association studies (using haplotypes or single SNPs) were implemented using a daughter design of 810 Saanen goats sired by 9 Artificial Insemination bucks genotyped with the goatSNP50 chip. A highly significant signal (-log10pvalue = 10.2) was associated with the “pink neck” phenotype on chromosome 11, suggesting the presence of a major gene. Highly significant signals for the “pink” phenotype were found on chromosomes 5 and 13 (-log10p values of 7.2 and, 7.7 respectively). The most significant SNP on chromosome 13 was in the ***ASIP* gene** region, well known for its association with coat color phenotypes. Nine significant signals were also found for both traits. The highest signal for each trait was detected by both single SNP and haplotype approaches, whereas the smaller signals were not consistently detected by the two methods. Altogether these results demonstrated a strong genetic control of the “pink” and “pink neck” phenotypes in French Saanen goats suggesting that SNP information could be used to identify and remove undesired colored animals from the breeding program.

## Introduction

Coat color has been a subject of scientific attention for many years and the inheritance of a coat color phenotype was already studied in different mammals in 1905 [1,2]. This trait is indeed one of the most identifiable when looking at an animal. For esthetic reasons, or if the aim is to have natural colored wool, the price of an animal (sheep, dogs, Alpacas…) may even depend on its coat color. Coat color also has a role in heat tolerance [3]. Moreover, coat color often forms part of the actual breed definition. In France, Saanen goats must have a uniformly white coat to comply with the breed standard (http://www.capgenes.com/IMG/pdf_Saanen_anglais.pdf).

**Fig 1:**
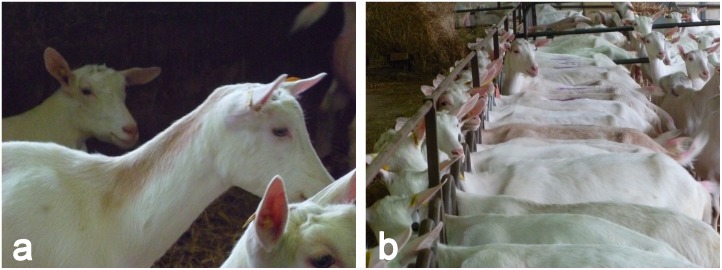
Coat color phenotypes (a) PINK NECK and (b) PINK in the French Saanen breed (Personal pictures).

The coat color of French goats is phenotyped by Capgenes, the organization in charge of the breeding program. Two distinct phenotypes for coat color defects have been registered in Saanen flocks: “PINK” (completely pink coat) and “PINK NECK” (see Fig 1). Males carrying one of these phenotypes and goats carrying the “PINK” phenotype are not used for mating which results in a substantial loss for the breeding program. To our knowledge, no investigation has been carried out on the genetic components of pink phenotypes in Saanen goats.

No fewer than 127 loci have already been identified to be associated with color in mammals [4] and at least 11 loci (Agouti, Albino, Australian Piedbald, Brown, Extension, Pigmented Head, Roan, Spotting, Sur Bukhara, Sur Surkhandarya and Ticking) affect coat color in small ruminants [5]. The genetic and physiological bases of pigment synthesis have been widely studied in mammals, particularly in mice [6,7]. However, the cellular and molecular events governing pigmentation patterning are still poorly understood and the factors controlling coat color remain complex. Two genes, Agouti (i.e. *ASIP*) and Extension (i.e. *MC1R*) are often highlighted because they balance the type of pigment produced [8–10]: the MC1R protein, with its usual ligand, leads to production of the dark pigment (eumelanin) whereas the secretion of ASIP, antagonist of the MC1R protein, causes a switch to light pheomelanin production. The dominant allele of the ASIP gene is commonly suggested to be responsible for the white color in Saanen goats [11,12]

The goat genetics context has changed since 2010 following sequencing of the goat genome [13] and the release of the goatSNP50 chip [14] developed by the International Goat Genome Consortium (IGGC), which now permits genome wide association studies (GWAS).

Our aim in this work was first to determine the genetic parameters of the two coat color traits, and then to perform a GWAS, based on goatSNP50 chip data, to identify the genomic regions responsible for “pink” and “pink necked” animals.

## Results

### Frequency of the pink and pink neck phenotypes in the national goat breeding program

The distributions and frequencies of each phenotype are presented in Table 1. The proportion of PINK (8.0%) is a little lower than PINK NECK (11.5%). These percentages are not negligible and mean that potentially one fifth of animals are discarded from the breeding program because of their coat color. This represents a considerable loss for the French national breeding program.

**Table 1:** Distribution of coat color phenotypes among 103 443 French Saanen goats scored in the performance control system with known genealogy and born between 2004 and 2010.

Phenotype	Number of animals	Percentage
PINK	8,285	8.0
PINK NECK	11,923	11.5
White	83,235	80.5

**Fig 2:**
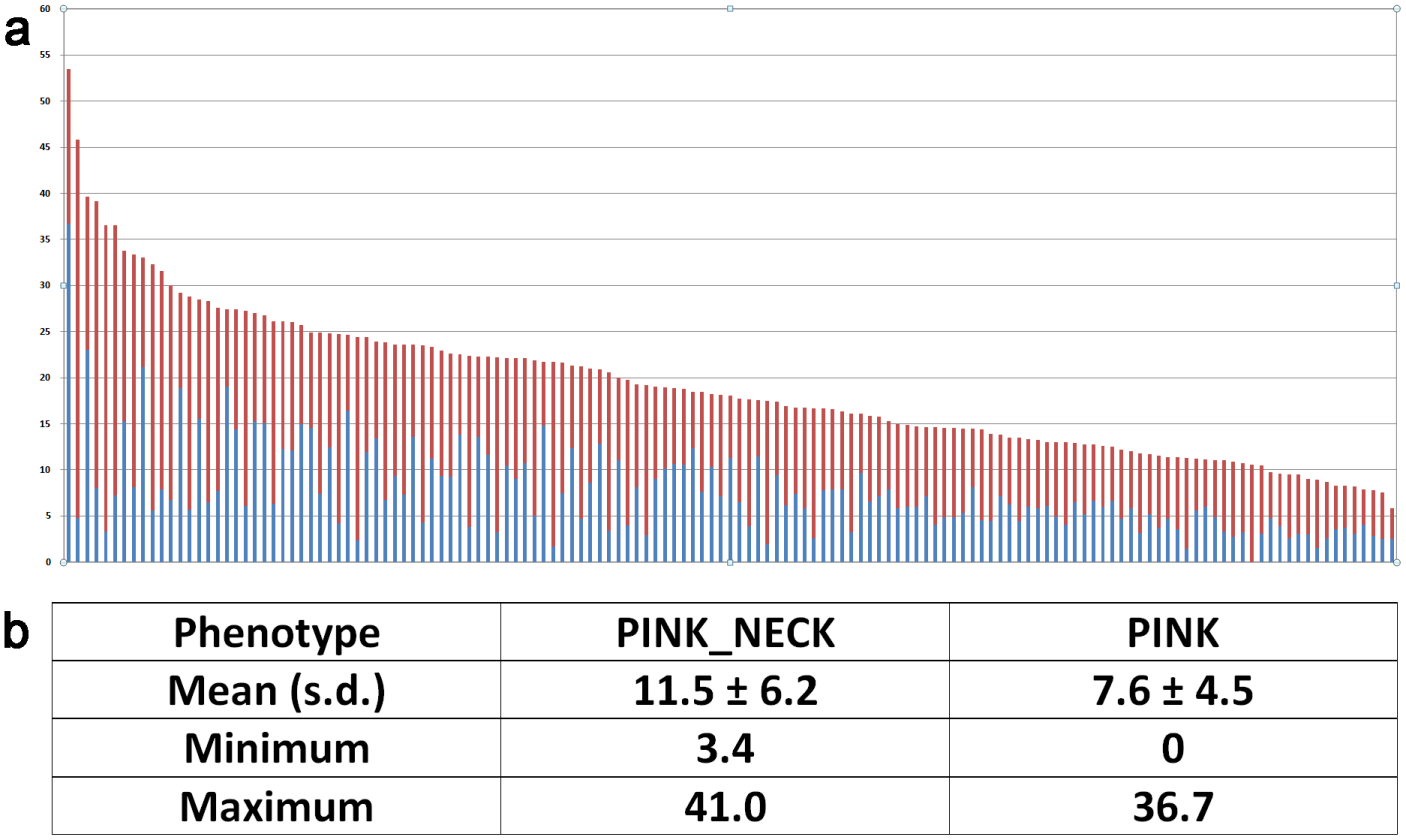
Distribution (a) and average values (Mean, Standard Deviation, Minimum and Maximum) (b) of the PINK_NECK and PINK frequencies by sire in the 143 half-sib families with more than 100 daughters. For a: Each column corresponds to one family (= a sire and all his daughters), with the proportion of PINK_NECK in red and the proportion of PINK in blue. Mean family size was 393 animals (standard deviation of 284 animals).

The average frequencies of PINK and PINK NECK animals in families with more than 100 daughters per sire were similar to those of the entire population (Fig 2), and were therefore considered representative. The frequencies of the colored phenotypes in the 143 families concerned were highly variable: in 14 families, less than 10% of daughters had colored phenotypes whereas in six families 35% were colored. The relative standard deviation of each phenotype was indeed slightly higher than 50%. The relative numbers of PINK and PINK NECK animals within the families were also highly variable, as seen in Fig 2. In some families most animals were PINK NECK whereas in others they were PINK. Some families had equal proportions of both types while one family had no PINK at all but only white or PINK NECK animals.

In our sample for the GWAS, the frequencies of PINK and PINK NECK were 9.5% (77 animals) and 10.3% (83 animals) respectively, similar to the national population frequencies.

### Genetic parameters

The estimated heritability for PINK NECK was 0.21 (standard error = 0.03) and a little higher for PINK i.e. 0.26 (s.e. = 0.03).

The possible genetic correlation between the two traits was then explored by calculating the correlation between the PINK and PINK NECK frequencies for families with more than 100 daughters. The resulting correlation of 0.03 suggested independence.

### Population stratification assessment

**Fig 3:**
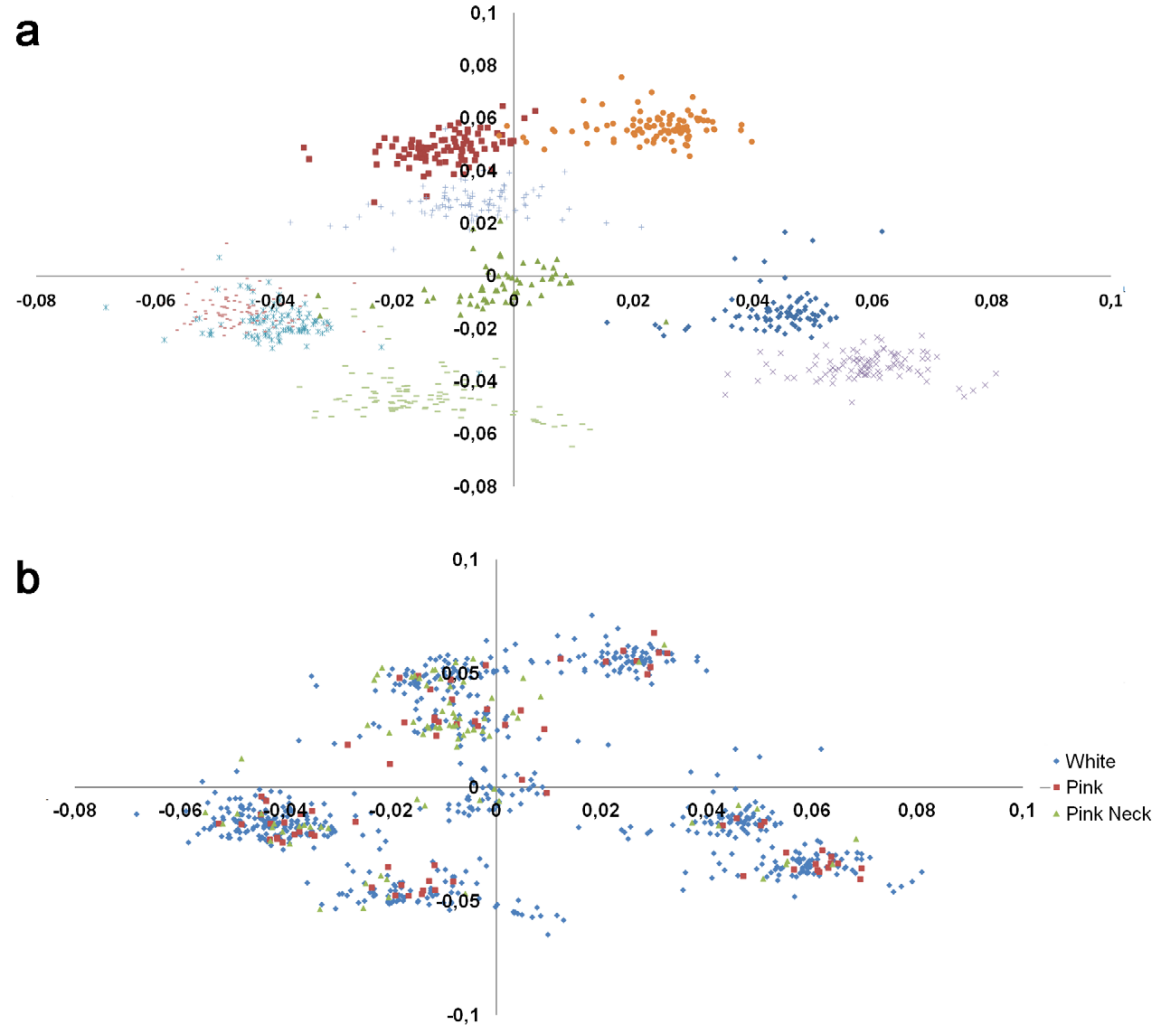
Population structure map drawn from the first two principal components a) by family with each symbol corresponding to one family b) by phenotype.

Results from a principal component analysis (PCA) based on SNPs information were then used to construct and plot the population structure and investigate the possibility of improving the subsequent analyses. As illustrated in Fig 3a, the population structure reflects the nine families in our daughter design. Fig 3b shows that the studied phenotypes are well distributed on the graph. As the GWAS used a relationship matrix to take family structure into account, no additional correction was made for the population stratification.

### Validation of the QQplots

**Fig 4:**
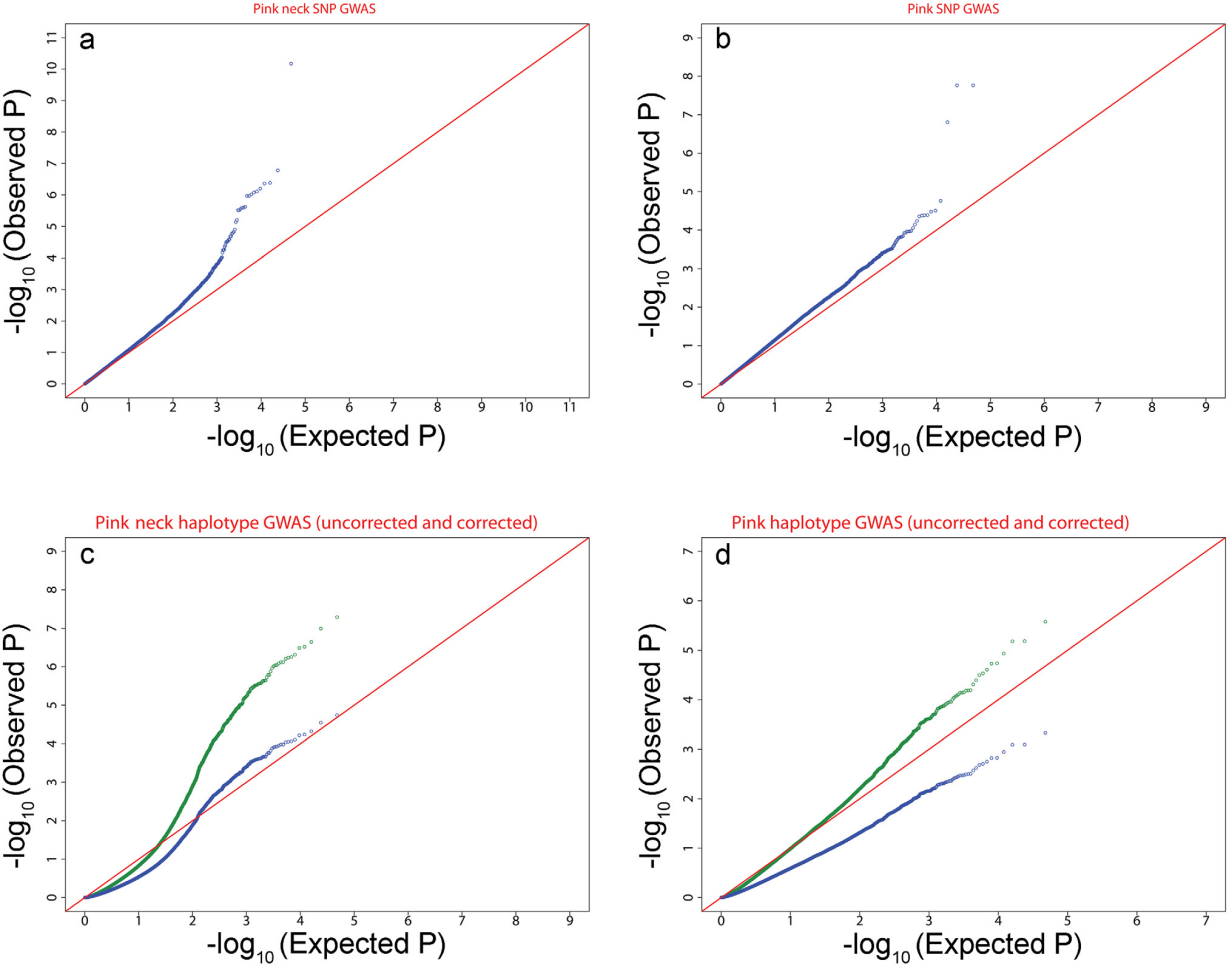
Quantile-Quantile plots for the different GWAS analyses, uncorrected (blue) and after correction (green, only in the case of the GWAS by haplotype for the PINK trait). a: PINK NECK trait for SNP GWAS; b: PINK trait for SNP GWAS; c: PINK NECK trait for Haplotype GWAS; d: PINK trait for Haplotype GWAS.

The quantile-quantile plots (QQplots) were then examined to determine the validity of the P value (Fig 4). For the SNP GWAS, the corresponding QQplots gave a reasonable confirmation of the distribution. Most SNPs followed the null hypothesis and there were only a few deviated SNPs (or a few positions in the case of the PINK NECK), indicating significant SNPs. No further correction was therefore applied. For the QQplots corresponding to the Haplotype GWAS, the distribution clearly deviated. A correction was applied using a regression coefficient (λ) calculated from the 90% less-deviating SNPs. The λ value used for the correction was 1.54 for PINK NECK and 1.67 for PINK.

### Significant SNPs—PINK NECK

**Fig 5:**
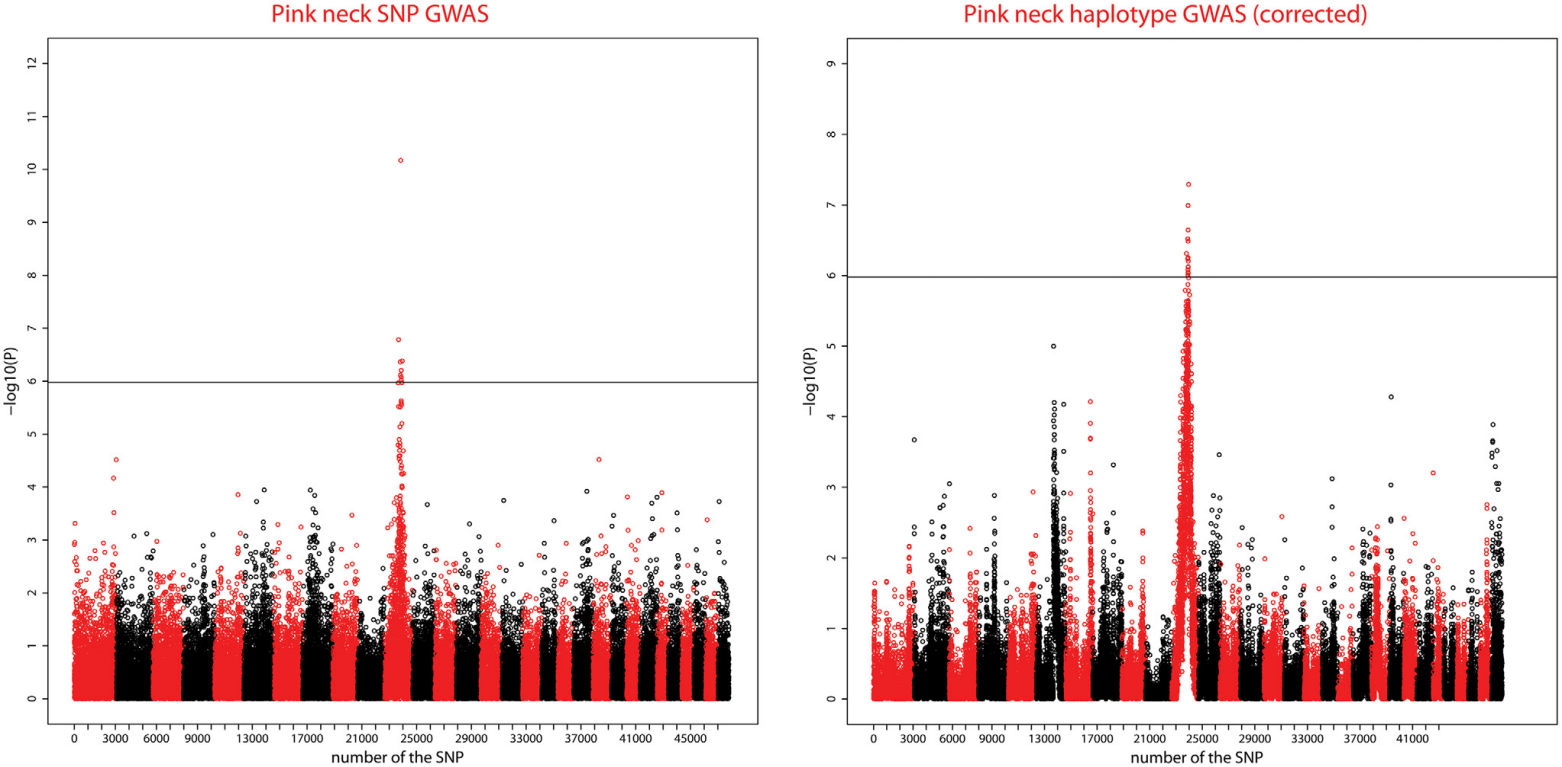
Genome-wide Manhattan plot of the SNP and Haplotype GWAS for the PINK_NECK trait. Manhattan plots show the combined association signals (−log_10_(p)) on the y-axis versus SNPs position in the goat genome on the x-axis and ordered by chromosome number. Black lines represent the 5% genome-wide threshold. Chromosomes are ordered from CHIR1 to CHIR29, the last one being the X chromosome.

Fig 5 shows the Manhattan plots for the SNP and Haplotype analyses. For the SNP GWAS, 24 chromosome-wise significant SNPs were detected on CHIR11, eight of which had genome-wise significance. The most significant SNP (snp15376-scaffold1630-140951), located at position 58.4Mb on CHIR11 had a −log_10_(P-value) of 10.2. One other chromosome-wise significant SNP was detected on CHIR 21 (see Table 2), at a lower significance level (- log_10_(P) = 4.5).

**Table 2:** List of all the significant signals from GWAS for PINK and PINK_NECK traits. The table gives for each signal: the corresponding trait and method, i.e. SNP (snp) or by haplotype (haplo), the chromosome number, the SNP where the maximum is reached and its position, the associated probability, the number of significant SNPs contributing to the peak, the interval of those SNPs and the significance level of the signal, i.e. *** for genome wise and ** for chromosome wise level calculated as explained in the materials and methods. The data refer to CHIR 1.0 assembly.

Trait/method	CHIR	SNPmax id	Physical position	P	Number of significant snps	Significant interval	Significance level
PINK_NECK snp	11	rs268248104	58.41 Mb	6.74 x 10^−11^	24	48.64–67.85 Mb	***
PINK_NECK snp	21	rs268264768	21.35 Mb	3.02 x 10^−05^	1	-	**
PINK_NECK haplo	6	rs268289590	67.96 Mb	5.63 x 10^−04^	1	-	**
PINK_NECK haplo	11	rs268250621	64.60 Mb	1.82 x 10^−05^	102	43.76–74.63 Mb	***
PINK snp	5	rs268259071	26.82 Mb	1.58 x 10^−07^	1	-	***
PINK snp	13	rs268239486	61.48 Mb	1.72 x 10^−08^	2	61.48–61.52 Mb	***
PINK snp	19	rs268260213	13.13 Mb	1.76 x 10^−05^	1	-	**
PINK snp	25	rs268245226	4.64 Mb	3.13 x 10^−05^	1	-	**
PINK haplo	7	rs268286829	16.49 Mb	1.49 x 10^−03^	1	-	**
PINK haplo	11	rs268265841	16.69 Mb	1.14 x 10^−03^	1	-	**
PINK haplo	11	rs268285720	57.32 Mb	8.04 x 10^−04^	1	-	**
PINK haplo	13	rs268239486	61.48 Mb	8.11 x 10^−04^	2	61.48–61.52 Mb	**
PINK haplo	22	rs268248034	40.19 Mb	2.39 x 10^−03^	1	-	**
PINK haplo	X	rs268247755	26.76 Mb	2.68 x 10^−03^	1	-	**
PINK haplo	X	rs268255768	47.58 Mb	1.79 x 10^−03^	1	-	**

In the haplotype-based association, the same peak was detected on CHIR 11 (Fig 5) with a higher density of significant positions along the entire chromosome. This peak comprised 102 chromosome-wise significant SNPs of which 15 were genome-wise significant. The maximum was reached at the position 64.6Mb with −log_10_(P) = 7.3.

The haplotype-based GWAS highlighted another signal on CHIR 6 but consisting of only one significant position and at the chromosome-wise level (see Table 2).

### Predictive power of the most significant SNP for PINK_NECK

The distribution of the animals, regarding their performance and genotype for the most significant SNP (snp15376-scaffold1630-140951), is presented in Table 3. The frequency of G, the allele associated with PINK NECK, was 15%. According to the model the estimated effect of this allele was 21%. The positive predictive value associated with PINK NECK and the G allele was 28%, the negative predictive value was 95%, sensitivity was 69% and specificity 77%.

**Table 3:** Distribution of the 733 Saanen goats by phenotype and genotype at the snp15376-scaffold1630-140951.

Genotype at snp15376-scaffold1630-140951	PINK_NECK	WHITE
AA	25	503
AG	49	136
GG	8	12

### Significant SNPs—PINK

**Fig 6:**
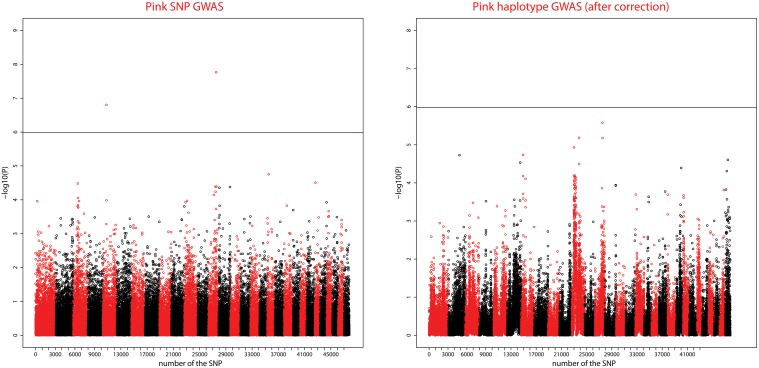
Genome-wide Manhattan plot of the SNP and Haplotype GWAS for the PINK trait. Manhattan plots show the combined association signals (−log_10_(p)) on the y-axis versus SNPs position in the goat genome on the x-axis and ordered by chromosome number. Black lines represent the 5% genome-wide threshold. Chromosomes are ordered from CHIR1 to CHIR29, the last one being the X chromosome.

Fig 6 shows Manhattan plots for the SNP and Haplotype analyses. For the SNP GWAS, five chromosome-wise significant SNP were detected on 4 CHIR (see Table 2). Two signals (CHIR 5 and 13) were genome-wise significant. The highest signal was on CHIR 13, with a maximum attained by two genome-wise significant SNPs (snp6524-scaffold1231-179272 and snp6523-scaffold1231-142303), which are adjacent SNP on the goatSNP50 chip, at position 61.5Mb, with −log_10_(P) = 7.7 for both. The other genome-wise significant SNP (located on chromosome 5) had a lower P-value (-log_10_(P) = 7.2).

In the haplotype-based association, eight SNPs on five chromosomes (CHIR 7, CHIR 11, CHIR 13, CHIR 22, and CHIR X) were significant at the chromosome-wise level (see Table 2) but none at the genome-wise level. The two significant SNPs on CHIR 13 detected with the SNP analyses were again present but the most significant positions were significant only at the chromosome-wise level (-log_10_(P) = 5.2 and 5.6). The Manhattan plot revealed another peak on chromosome 11 with two chromosome-wise significant SNPs in the same position as the signal found for PINK NECK.

### Predictive power of the most significant SNPs for PINK

The two significant SNPs on CHIR 13 exhibited a perfect LD and gave redundant information. The effect explained by the SNPs was high (36%) but due to the low frequency of the associated allele (2%), may have been overestimated. The association between genotypes and phenotypes for the snp6524-scaffold1231-179272 can be seen on Table 4. The positive predictive value was 51%, the negative predictive value 91%, sensitivity was 19% and specificity was 98%.

**Table 4:** Distribution of the 727 Saanen goats by phenotype and genotype at the snp6524-scaffold1231-17927.

Genotype at snp6524-scaffold1231-17927	PINK	WHITE
AG	15	14
GG	62	636

## Discussion

### PINK: a candidate gene

The goat genome map and annotation, available in http://www.ncbi.nlm.nih.gov/genome?term=capra%20hircus, were scrutinized for the most significant positions for the two traits.

For the PINK trait, the two SNPs with the highest signal, on CHIR 13, were located near the Agouti Signaling Protein, *ASIP* gene (position 61.7 Mb on CHIR 13), a strong functional candidate. This gene is well known for its action in coat color control and has already been shown to be associated with coat color phenotypes in various mammals [15,16], including goats [17]. Molecular mechanisms have been described in mice [18–20] in which the ASIP gene encodes a paracrine signaling protein that binds to and inhibits signaling through the melanocortin-1 receptor (MC1R). This inhibition of MC1R signaling results in decreased cAMP intracellular concentrations leading to the induction of follicular melanocyte which synthesizes pheomelanin, a yellow/red pigment, instead of eumelanin, a black/brown pigment.

A large number of alleles (about 20) have been described in goats for the *ASIP* gene, although some probably overlap [12]. Adalsteinsson et al. [11] proposed the existence of 11 alleles. The allele responsible for white color in the Saanen breed always seems to be considered as the most dominant [11,12]. The presence of Copy Number Variation (CNV) at the Agouti locus has been confirmed and the number of copies might be responsible for the various alleles, especially the dominant white allele in the Saanen breed [12,21,22] as already suggested in [23].

### PINK NECK: a large zone without an obvious candidate gene

The main region on CHIR 11 associated with PINK NECK has a large confidence interval: 20Mb and 30Mb in width according to the SNP GWAS and haplotype GWAS, respectively. When we concentrated on the 9Mb region around the most significant positions obtained with the two methods (between 57 and 65Mb), it was found to include no less than 44 genes. These genes are listed in S1 Fig. None of these occur in the list of 378 loci known to be associated with color phenotypes in mice or in their human and zebrafish homologues (http://www.espcr.org/micemut/). This result emphasizes the need for further detailed analyses of the region to allow fine mapping and identification of the causal gene and mutation.

### Potential use of significant SNPs as predictor

Although the causal mutations of the two traits have not yet been identified, breeders can use associated SNPs as predictors and this option would be feasible for PINK NECK. The highest SNP would indeed provide a good predictor for avoiding PINK NECK animals and could be used to choose between young male twins at the beginning of the selection process or to avoid matings with females at risk.

For the PINK trait, the negative predictive value indicates that the SNP on CHIR 13 would not be a good predictor and the proportion of PINK animal would remain around its current level (8%) even after selection on this marker. Further investigations need to be done at the molecular level to find the possible causal mutation or at least a better predictor.

### Phenotypes and genotype confusion

As mentioned previously, the main loci involved in the PINK NECK and PINK traits, and thus the genetic determinism, are different. However, completely pink animals may carry the pink neck allele or genotype, so some confusion is possible. Indeed, we can assume that the significant SNPs for PINK on the CHIR 11 according to the haplotype-based association (and strongly associated with PINK NECK) are due to the presence of animals identified as completely pink but also carrying pink neck alleles.

Besides the confusion due to this partial inclusion, defining the animal’s coat colors is not always straightforward. The color intensity in PINK NECK and PINK animals is highly variable. Also, depending on the light in the barn or if the animals are dirty, animals with a slight coloration might be identified as white. Also the color can often be more intense on the neck than on the rest of the body, so slightly PINK animals may be phenotyped as PINK NECK. Some genetic markers would be useful for breeders to avoid possible confusion. It would be interesting in future to record color intensity for further and complementary analyses.

### SNP versus haplotype analyses

One assumption in SNP GWAS is that the markers are independent and not accounting for the potential linkage disequilibrium (LD) between SNPs. In comparison, the Haplotype GWAS is based on phased haplotype clusters in association and accounts for the potential linkage disequilibrium between markers.

Based on both simulated and real barley data, Lorenz et al. (2010) concluded that superiority of one over the other method to detect QTLs depends on the population history and architecture of traits and recommend use of haplotype methods as a complement to SNP GWAS. Strength of the SNP methods include the fact that errors in map order would have no effect on the single SNP analysis but may lead to improper inference of haplotype alleles. Also, Haplotype GWAS tests are based on chi-square distribution of more degree of freedom than for single SNPs, so that for the same p-value the likelihood ratios need to be higher to reach the significance thresholds [24]. The multiplicity of alleles combinations of haplotype GWAS, however, is an advantage to more likely detect a complex signal, e.g. more than two alleles at the causal mutation or two possible mutations in the same gene.

Our results showed that a few significant regions were detected by both association methods, but that each method found additional SNPs which were not detected by the other method. Some could be false positives, but in view of the QQplots, the haplotype-based association was not obligatorily more accurate in our case than the SNP based one. Moreover, the robustness of haplotype-based association is highly affected by the accuracy of haplotype reconstruction, which depends on the family structure of the sample.

The observed differences in the results are more likely to stem from the variation in linkage disequilibria between significant SNP and their neighbors. The SNPs on chromosome 11 seem to be closely linked with each other, which is why the signal for PINK NECK appeared denser and had a more highly significant position according to the haplotype-based analysis. Conversely, the two significant SNPs for PINK were not as highly significant in the haplotype-based analysis because they were the only two associated with the phenotype and their significance was diluted by neighboring SNPs. In fact, these two SNPs are located just before a large gap of 300,000 bp in the coverage of chromosome 13 by the chip (probably due to a CNV), so that, at least on one side, their neighbors are unusually distant. As different types of signals were more easily detected by one or other method, we recommend using both.

In summary, this first heritability estimation provided evidence of substantial genetic variation for two undesirable coat color phenotypes in Saanen goats and the large amount of genotyping data was used to localize significant genomic regions associated with PINK or PINK NECK. Whereas the main signal found for PINK is probably due to an already well-known gene (*ASIP*), the evidence of a major region for PINK NECK is new with regards to known color loci. This may be due to the different origins of the two phenotypes: the PINK coloration might be derived from an ancient inclusion of Alpine blood in the Saanen breed whereas PINK NECK seems to be specific to the Saanen breed. The mode of inheritance of these two traits is not straight forward. They are not monogenic dominant as at least one parent of each colored animal would then itself be colored, but this was not the case. They are not monogenic recessive either because this would imply that all the sires carried at least one copy of the mutant allele, there being at least one colored daughter in every family, which is very unlikely as the frequency of the phenotypes was not high. Their determinism is probably due to reduced penetrance and an effect of additional loci, other than those mentioned here, cannot be excluded. The results of this work can be used, short-term, for SNP-based selection to avoid PINK NECK. In view of the heritabilities of the two traits, the results can also be used to calculate the estimated breeding values and efficiently avoid both PINK and PINK NECK. This work emphasizes the need for further detailed analyses of the genomic regions identified to evidence the causal mutations and/or offer better genomic predictors.

## Materials and Methods

### Population resources and phenotypes

The pedigrees and phenotypes used in this study were extracted from the French national genetic database and the animals were Saanen goats involved in the national breeding program.

All females scored by Capgenes, with known genealogy and born between 2004 and 2010, were used for the phenotype frequency and heritability estimations. Flocks with fewer than 10 animals and flocks without any colored animal were discarded. In total, 103,443 animals were included in this dataset.

The GWAS data came from the subset of Saanen goats involved in a daughter design implemented in 2009 and 2010 as part of the national "Phénofinlait" (www.phenofinlait.fr) and the EU "3SR" (www.3srbreeding.eu) projects. The design consisted of 936 goats from 88 herds that were sired by nine different artificial insemination (AI) bucks. The nine bucks were selected to be both representative of the genetic diversity of the whole population and to maximize the genetic diversity between families, from the widely used AI bucks that had large numbers of daughters in commercial farms between 2009 and 2010.

For these two datasets, animals were phenotyped during their first or second lactation by official classifiers from the breeders’ association Capgènes (Mignaloux Beauvoir, France). The information about coat color is qualitative: totally white (in agreement with the breed definition), pink neck (pink hair on the neck, see Fig 1a) or pink (completely pink or with pink spot(s) between shoulder and tail, see Fig 1b). As the measurements were exclusive, and to take into account the hypothesis of dominance of the white color, the traits were analyzed as follows: PINK = pink in contrast to white (pink neck excluded) and PINK NECK = pink neck in contrast to white (pink excluded). All AI bucks included in the genetic parameter estimation and in the GWAS were white and only partial information about the mothers was available.

### Phenotype frequencies

Phenotype frequencies were calculated using the SAS^®^ V9.2 software. The phenotype frequencies in the dataset used for the GWAS were also calculated in order to compare this sample with the whole population.

The distribution of the PINK NECK and PINK frequencies across families was obtained by only including the 143 largest half-sib families, those with more than 100 daughters sired by a given AI buck, in the calculations of frequencies by family. All 143 sires were different.

### Estimation of heritability

Variance components for the two binary variables (PINK NECK and PINK) were estimated with the following animal logistic model with mixed effects using ASReml 3.0 Software [25]:

Logit(p)=Wi+Za+e (1)

Where $log\text{it}(\text{p})=ln[\frac{P(Yni=1)}{1-P(Yni=1)}]$ and p = P(Y_ni_ = 1) denotes the probability of goat n having the colored phenotype with the vector of fixed effects i, including flock of birth and year of birth; a is the random genetic animal effect; W and Z are the incidence matrices and e is the residual effect.

The pedigree file consisted of four generations including 171,895 animals. The heritability of the traits was estimated from the ratio of the animal variance component to the sum of the animal variance component and the residual variance.

### Ethics Statement

DNA samples for this study came from France and are stored at LABOGENA. Neither sperm collection nor blood sampling was performed specifically for this study. Sperm was collected from bucks by Capgenes, with the authorization from the DGAL (Direction Générale de l'ALimentation) FR CC 860. Sperm collection was done by Artificial Insemination stations, and we used extra doses from this collection. Blood samples were taken at commercial farms. The animals were not part of any experimental design but were sampled by veterinarians and/or under Veterinarian supervision for routine veterinary care; extra samples were requested when blood sampling took place.

### GWAS

#### Quality control of SNPs

All 936 goats and nine bucks were genotyped using the Illumina goatSNP50 chip [14]. DNA extraction from blood samples and genotyping were performed at the Laboratoire d’Analyses Génétiques pour les Espèces Animales, Jouy en Josas, France (LABOGENA; www.labogena.fr). **Quality control** included SNP call rate (>99%), animal call rate (>98%), minimum allele frequency (>2%), Hardy Weinberg equilibrium (P-value above 10^−6)^ and pedigree inconsistency removal. A total of 125 goats were discarded because of pedigree inconsistencies or low animal call rates. After these controls, 810 Saanen goats, including 83 pink neck and 77 pink animals, and their nine fathers (all white) remained. After editing, a total of 49,574 out of 53,347 synthesized SNPs were validated for further analyses. The marker order and positions were based on the caprine Assembly CHIR_1.0 (http://www.ncbi.nlm.nih.gov/assembly/GCF_000317765.1) and can be downloaded from the following link: http://www.goatgenome.org/data/capri4dbsnp-base-CHI-OAR-UMD.csv.

#### Population stratification assessment

Population stratification was assessed by calculating the genetic relationship matrix from all SNPs, and the two principal components, using the GCTA software [26]. The first principal component values were plotted against the second principal component values to identify potential clustering among the white and colored animals.

#### Genome wide association analyses

GWAS were carried out for each trait (PINK, and PINK NECK) with two models.

The first model was a SNP GWAS: the effect of each SNP was tested individually from the following logistic model with mixed effects:

Logit(p)=Wb+Za+e (2)

Where $log\text{it}(\text{p})=ln[\frac{P(Yn=1)}{1-P(Yn=1)}]$ and p = P(Y_n_ = 1) denotes the probability of goat n having the colored phenotype; *b* is the vector of fixed SNP effect (allele effect of the SNP); *W* is the incidence matrix of *b* corresponding SNP effect to individuals; *a* is the random genetic animal effect (polygenic effect) where *a* is assumed to follow a ~ N(0, Aδ_a_^2^) and A is the relationship matrix based on pedigree information. *Z* is the incidence matrix of *a* accounting for pedigree relationship structure among individuals and *e* is the random residual effect following *e* ~ N(0, Iδ_e_^2^) where I is the identity matrix. Only the four most recent generations of the pedigree were used in this analysis.

These calculations were done with restricted maximum likelihood using ASReml Software [25]. After calculating each SNP effect a T-test was performed to calculate the −log_10_(p-value). Because of the test multiplicity, a Bonferroni correction of α = 5% was applied for both genome-wise and chromosome-wise thresholds (Significance threshold = − log_10_(α/number of SNPs)). SNPs with P<1.04 x 10^−6^ were considered to be significantly associated at the genome-wise level.

The second model used haplotypes and data were phased using PHASEBOOK [27]. The PHASEBOOK method is a software package designed to obtain phased haplotypes in a population with high relationships. Haplotypes were first reconstructed based on familial information (Mendelian segregation rules and linkage information) using LinkPHASE. The gaps between the reconstructed elements were then filled in by applying a Hidden Markov Model from BEAGLE. Parameters recommended by Druet and Georges [27] were then used with different software: LinkPHASE was run with the threshold probability value to attribute parental origin set at 1.0. DAGPHASE was then run to attribute randomly missing alleles before the use of BEAGLE which, used iteratively with DAGPHASE, constructed optimal directed acrylic graphs (DAG) and improved those DAG at every iteration. BEAGLE was used with parameters scale = 1.0 and shift = 0.0 and with 10 iterations. The outputs of BEAGLE included haplotypes and the hidden states used to construct them. The hidden cluster effect was defined for each SNP position and ranged from 1 to 132.

The model used for this haplotype-based association was identical to the first model except that the haplotype effect (or hidden cluster effect) was fitted instead of a SNP in the model.

Logit(p)=Wβ+Za+e (3)

Where $log\text{it}(\text{p})=ln[\frac{P(Yn=1)}{1-P(Yn=1)}]$ and p = P(Y_n_ = 1) denotes the probability of goat n having the colored phenotype; *β* is the vector of haplotype effect (hidden cluster effect); *W* is the incidence matrix of *β* corresponding haplotype effect to individuals (the two haplotypes per animal and position were fitted as two related fixed effects so that the same haplotypes provided the same effects); *a* is the random genetic animal effect (polygenic effect) and *Z*, *a* and *e* are the same as in model [2]. The pedigree used is the same.

Restricted maximum likelihood (REML) was applied to solve the model using ASReml [25]. The haplotype effects of all haplotypes were calculated and an F-test was performed for significance and a Bonferroni correction was applied at α = 5%.

### Validation of test-statistics with QQ-plot and potential correction

Quantile-Quantile plots (QQ-plots) were produced for each trait and each model to check the distribution of the test-statistics and detect the degree of fit of the model for this trait. In the case of deviation on the QQ-plot (apparent underestimated or overestimated p-values in the distribution), the tests were regressed from the 90% less deviated SNPs to obtain the theoretical P distribution so that the regression coefficient on those SNPs was 1.

### Evaluation of SNPs as a predictor for traits of interest

The value of the best SNPs found by GWAS analyses as a predictor was evaluated from the positive predictive value (proportion of animals with the associated allele who actually have the considered colored phenotype), the negative predictive value (proportion of animals without the associated allele who actually do not have the considered phenotype), the sensitivity (proportion of animals with the associated allele among all the animals with the considered phenotype) and the specificity (proportion of animals without the associated allele among those without the considered colored phenotype).

## Supporting Information

S1 Fig List of the 44 genes included between 57 and 65 Mb on the CHIR 11, based on the NCBI annotation. (TIF)
