# Correction: Genome Wide Association Study Identifies New Loci Associated with Undesired Coat Color Phenotypes in Saanen Goats

**DOI:** 10.1371/journal.pone.0186029

**Published:** 2017-10-05

**Authors:** Pauline Marie Martin, Isabelle Palhière, Anne Ricard, Gwenola Tosser-Klopp, Rachel Rupp

The following information is missing from the Funding section: PMM received a doctoral research grant from “Région Midi-Pyrénées”, Animal Genetics Department from INRA and GENOMCAP research program including INRA, APIS-GENE, ALLICE (formerly UNCEIA), CAPGENES and FCEL.
