# Supplementary figures and images for: Genome Wide Association Study Identifies New Loci Associated with Undesired Coat Color Phenotypes in Saanen Goats
Source: PLoS One. 2016 Mar 31;11(3):e0152426. doi: 10.1371/journal.pone.0152426 (PMC4816504; doi:10.1371/journal.pone.0152426)

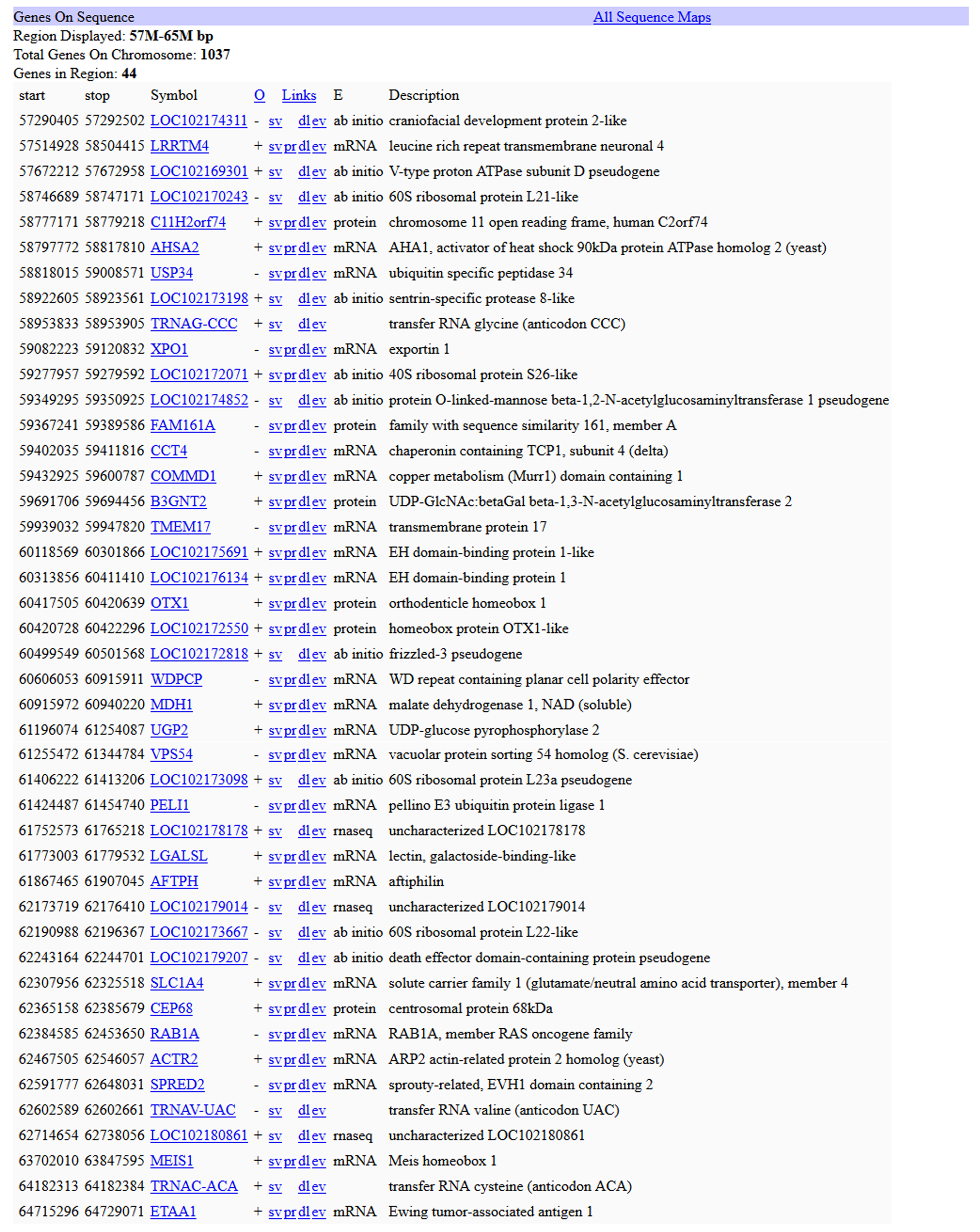

Supplement: S1 Fig — (TIF) [file pone.0152426.s001.tif]
